# Blood-Corpuscles and Spermatozoa of the Camelidæ

**Published:** 1843-07

**Authors:** 


### Blood-corpuscles and Spermatozoa of the Camelidæ.

After M. Mandl had discovered the oval shape of the blood-discs of the dromedary and paco, Mr. Gulliver ascertained that those of the vicugna and llama have the same form, (see Appendix to Gerber's Anatomy, pp. 10 and 43.) To complete the history of the blood-corpuscles of this family, Mr. Gulliver has lately given an account of those of the camel (*Camelus Bactrianus*), from which it appears that their average long diameter is $\frac{1}{3123}$d, and their short diameter $\frac{1}{5876}$th of an English inch. (See Proceedings of the Zoological Society of London, Part x. p. 191.)

Thus the corpuscles of this animal are elliptical; and their form, as far as we yet know, is confined to the camelidæ among the mammalia, a very curious anomaly, especially as Mr. Gulliver's observations have shown that even the blood-discs of the marsupialia have the usual circular figure. (See Willis's translation of Wagner's Physiology, Part II. p. 234, *note.*)

At the meeting of the Zoological Society, April 11th, 1843, Mr. Gulliver read an account of the spermatozoa of the camel, confirming that which he had previously given of those of the dromedary, (Proc Zool. Society, Part X. 1842, p. 355,) namely, that they are of the same type as the corresponding animalcules of other mammals. This fact is curious, because, from the singular correspondence of form between the blood-corpuscles of the camelidæ and the corpuscles of the oviparous vertebrata, it might have been supposed that a similar affinity would exist between the essential elements of the semen of these animals.

